# Cold Agglutinin-Mediated Hemolytic Anemia Preceding the Diagnosis of Acute Myeloid Leukemia: A Case Report and Literature Review

**DOI:** 10.7759/cureus.106577

**Published:** 2026-04-07

**Authors:** Muhammad Ali Tariq, Motaz Almahmood, Javaria Mushtaq, Jason Incorvati

**Affiliations:** 1 Internal Medicine, Tower Health Medical Group, Phoenixville, USA; 2 Cell Biology and Neurosciences, Rowan University, Stratford, USA; 3 Hematology and Medical Oncology, Fox Chase Cancer Center, Norristown, USA

**Keywords:** acute myeloid leukemia (aml), autoimmune hemolytic anemia (aiha), bone marrow biopsy, cold agglutinin, cold agglutinin syndrome, complement-mediated hemolysis, direct antiglobulin test (dat), rituximab

## Abstract

Cold agglutinin syndrome (CAS) is a rare complement-mediated autoimmune hemolytic anemia that is usually associated with infection, autoimmune disease, or lymphoproliferative disorders and is only rarely linked to acute myeloid leukemia (AML). We report a 78-year-old woman who presented with fatigue, macrocytic anemia, reticulocytosis, elevated lactate dehydrogenase, low haptoglobin, indirect hyperbilirubinemia, a direct antiglobulin test positive for C3 and negative for IgG, and a cold agglutinin titer of 1:320. She was treated with rituximab, after which the titer declined to 1:5; however, progressive anemia developed, with hemoglobin falling to 6.5 g/dL, and peripheral blood findings evolved to show left-shifted myeloid maturation with circulating blasts. Flow cytometry demonstrated myeloblasts comprising 14% of analyzed cells, and bone marrow biopsy confirmed AML with 61% blasts and a U2AF1 mutation. Published reports of AML presenting with clearly documented cold agglutinin-mediated hemolysis are exceedingly rare. This case underscores that apparent serologic improvement in CAS does not exclude evolving marrow pathology and that worsening cytopenias, persistent macrocytosis, or new peripheral blasts should prompt timely marrow evaluation.

## Introduction

Cold agglutinin syndrome (CAS) is a form of autoimmune hemolytic anemia in which cold-reactive IgM antibodies bind erythrocyte antigens at lower temperatures, producing red blood cell agglutination, complement activation, and hemolysis [1,2]. CAS is typically secondary to infection, autoimmune disease, or lymphoid malignancy rather than to myeloid neoplasms [1,2].

The overlap with acute myeloid leukemia (AML) appears to be distinctly uncommon. In a multinational observational study of 232 patients with cold agglutinin disease (CAD), only two patients (0.9%) later developed AML [2]. Because cold agglutinin-mediated hemolysis is not typically associated with AML, the initial hemolytic diagnosis may narrow the differential diagnosis and delay recognition of underlying marrow pathology. We report a case of cold agglutinin-mediated hemolytic anemia that preceded the diagnosis of AML.

## Case presentation

A 78-year-old woman with a history of grade II invasive ductal carcinoma, who had been on long-term hormonal therapy with letrozole 2.5 mg daily for the past four years following definitive surgery, presented for evaluation of worsening fatigue. She had previously been followed by hematology, and her hemoglobin had remained stable at approximately 12 g/dL until this presentation.

Repeat laboratory testing obtained by her breast oncologist showed hemoglobin 8.1 g/dL, hematocrit 23.9%, mean corpuscular volume 108 fL, white blood cell count 11 × 10^9^/L, and platelet count 73 × 10^9^/L. Further evaluation demonstrated biochemical hemolysis and a C3-positive direct antiglobulin test. Table 1 summarizes the laboratory evaluation.

**Table 1:** Laboratory evaluation EBV: Epstein-Barr virus; CMV: cytomegalovirus

Parameter	Result	Reference range
Hemoglobin (g/dL)	8.1	12.0-16.0
Hematocrit (%)	23.9	36-48
Mean corpuscular volume (fL)	108	80-99
White blood cell count (×10^9^/L)	11	4.0-10.8
Platelet count (×10^9^/L)	73	150-450
Reticulocyte count (%)	6.0	0.5-2.0
Lactate dehydrogenase (U/L)	1,172	120-246
Haptoglobin (mg/dL)	<10	30-200
Total bilirubin (mg/dL)	2.8	0.3-1.2
Direct bilirubin (mg/dL)	0.3	0.2-0.4
Direct antiglobulin test	C3 positive; IgG negative	Negative
Cold agglutinin titer	1:320	<1:32 negative; >1:64 elevated
EBV antibodies	Negative	Negative
CMV antibodies	Negative	Negative
HIV-1/2 Ag/Ab	Negative	Negative
Hepatitis B virus screening	Negative	Negative
Hepatitis C virus antibody	Negative	Negative
Complement C3/C4	Normal	Within reference range
Antinuclear antibody	Negative	Negative
Thyroid-stimulating hormone (mU/L)	4.1	0.4-4.0
Folate (ng/mL)	9	2-20
Vitamin B12 (pg/mL)	420	200-950

Cold agglutinin testing demonstrated an elevated titer of 1:320, supporting a diagnosis of CAS. She was treated with rituximab 375 mg/m^2^ intravenously once weekly for four weeks. Computed tomography of the chest, abdomen, and pelvis showed no evidence of recurrent or metastatic solid malignancy.

**Figure 1:**
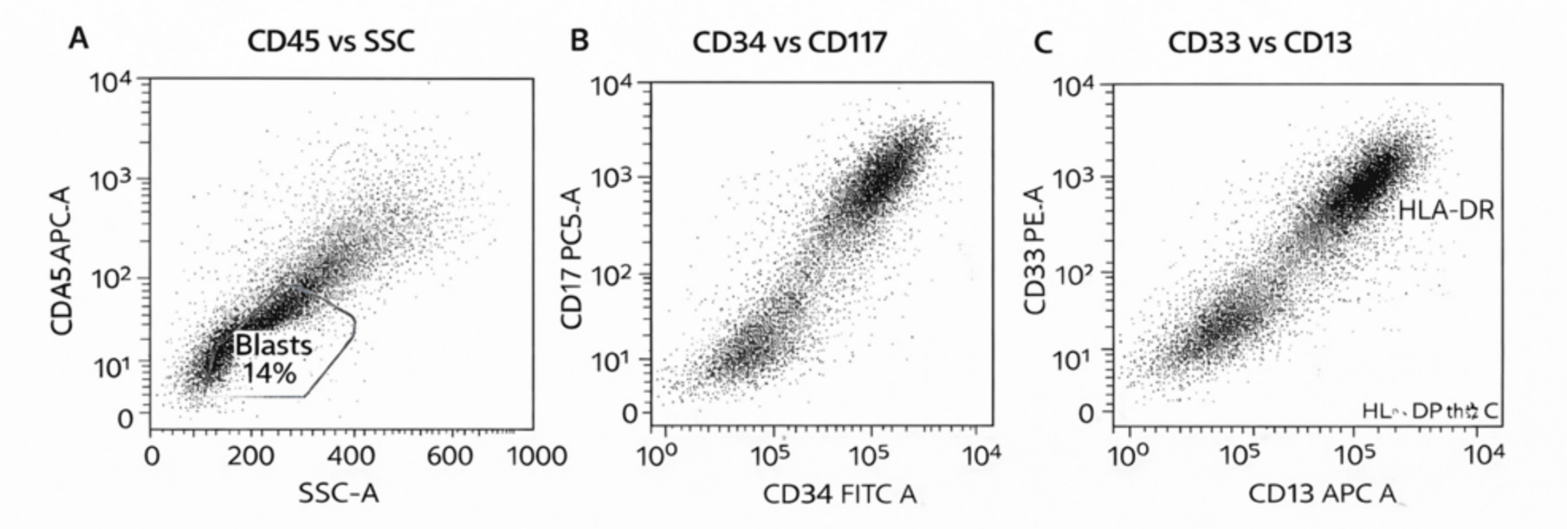
Flow cytometry identified a blast population comprising 14% of analyzed cells. The blast population was CD45-dim with low side scatter and expressed CD34, CD117, CD13, CD33, and HLA-DR

After rituximab, progressive anemia developed, with hemoglobin declining to 6.5 g/dL. Repeat cold agglutinin testing showed a decline in titer from 1:320 to 1:5 despite worsening anemia. Repeat studies showed left-shifted myeloid maturation with circulating blasts on the manual differential. Flow cytometry demonstrated a blast population comprising 14% of analyzed cells (Figure 1). Because of symptomatic anemia in the setting of CAS, she received one unit of warmed leukoreduced packed red blood cells, with compatibility testing and transfusion performed using warming precautions.

**Figure 2:**
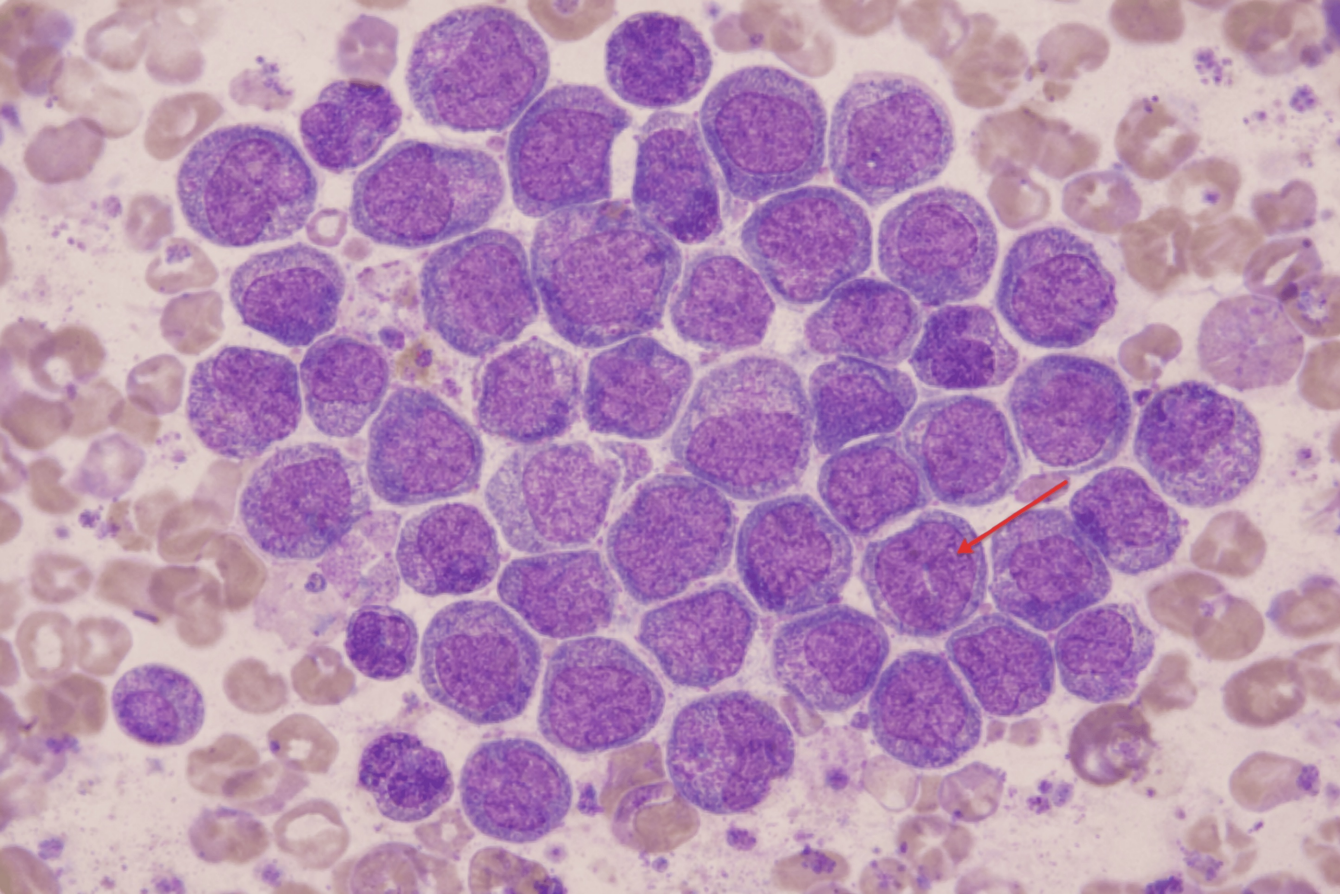
Bone marrow aspirate demonstrating numerous blasts; the arrow highlights a representative myeloblast

**Figure 3:**
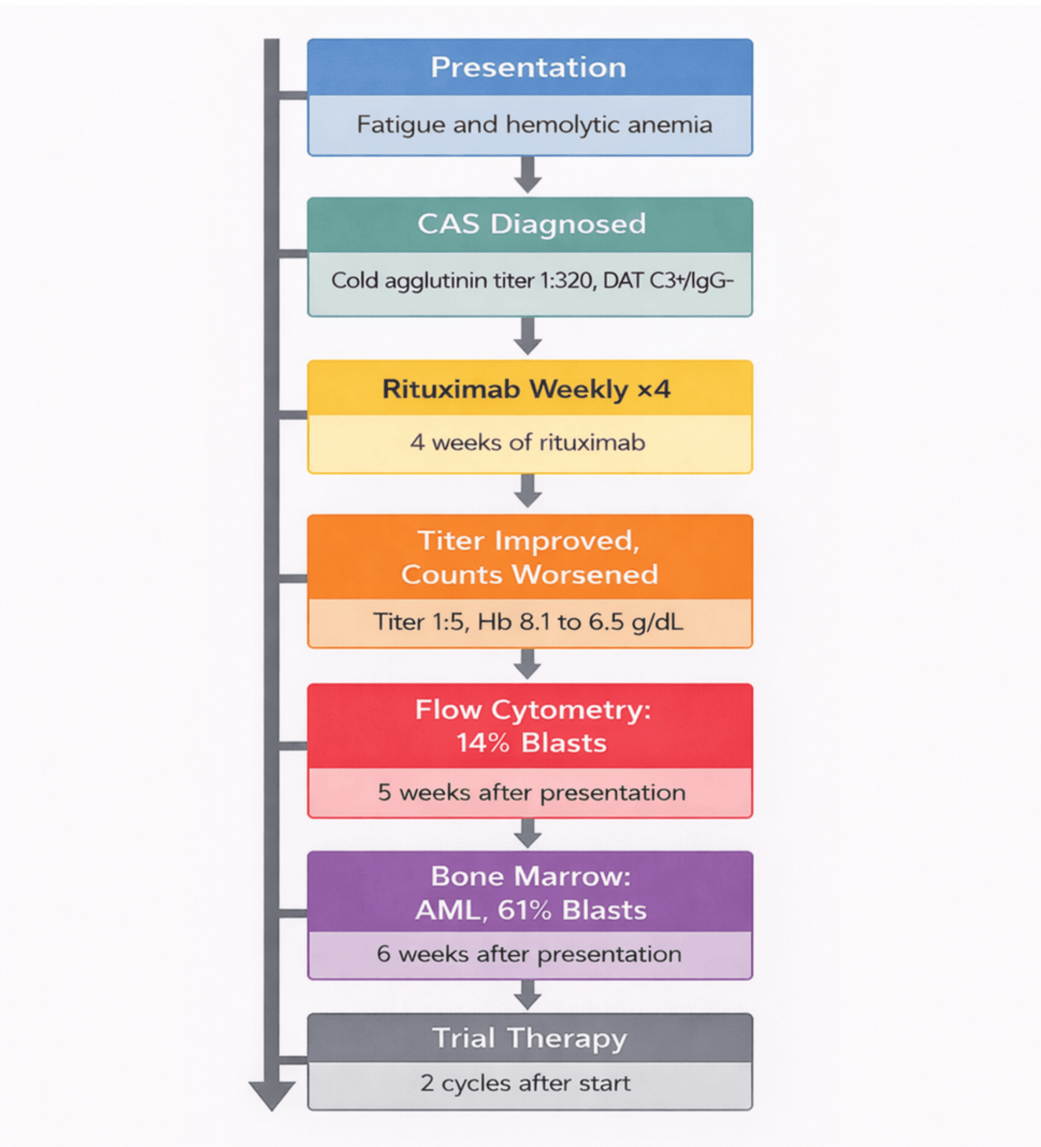
Timeline of key clinical events CAS: cold agglutinin syndrome; DAT: direct antiglobulin test; AML: acute myeloid leukemia

Given the discordance between improving serologic markers and worsening blood counts, she underwent a bone marrow biopsy to evaluate for an underlying marrow process. Bone marrow examination demonstrated AML with 61% blasts (Figure 2). Flow cytometry identified an abnormal blast population comprising 14% of analyzed cells that was CD45-dim with low side scatter and expressed CD34, CD117, CD13, CD33, and HLA-DR. Conventional cytogenetic analysis showed a normal female karyotype, 46,XX(20). Next-generation sequencing identified multiple mutations, including U2AF1, and the leukemia was classified as AML, myelodysplasia-related. By the European LeukemiaNet (ELN) 2022 criteria, this corresponded to adverse-risk AML. Following the diagnosis, she was enrolled in a clinical trial evaluating azacitidine, venetoclax, and the investigational anti-CD47 monoclonal antibody magrolimab. After two cycles of therapy, she developed severe septic shock and died despite supportive care. A timeline of key clinical events from the initial presentation of CAS to the diagnosis and treatment of AML is illustrated in Figure 3.

## Discussion

This case is notable because the initial laboratory picture strongly supported cold agglutinin-mediated hemolysis, and the elevated cold agglutinin titer provided a plausible explanation for the patient’s anemia. However, the subsequent clinical course was not fully explained by CAS alone. Importantly, the baseline combination of macrocytic anemia and thrombocytopenia also raised concern for an underlying marrow disorder, even before overt peripheral blasts became apparent. Apparent serologic improvement following rituximab therapy did not translate into hematologic recovery; instead, the anemia worsened, and new myeloid abnormalities emerged in the peripheral blood. This discordance was the key clue that prompted bone marrow evaluation and ultimately led to the diagnosis of AML. Throughout this report, we use the term CAS rather than CAD because the hemolysis appeared secondary to an underlying disorder rather than representing primary clonal CAD [1,2].

Published AML-associated immune hemolysis is uncommon, and clearly documented cases of cold agglutinin-mediated hemolysis appear to be exceptionally rare. The pathophysiologic link between AML and cold agglutinin-mediated hemolysis remains incompletely understood but is likely multifactorial. CAS is a complement-mediated autoimmune process driven by cold-reactive IgM antibodies that bind erythrocytes and activate the classical complement pathway [1,2]. In the context of AML, one possible mechanism is immune dysregulation associated with clonal hematopoiesis, which may promote aberrant B-cell activation and autoantibody production. Alternatively, CAS may represent a paraneoplastic phenomenon, in which cytokine-mediated immune activation and loss of immune tolerance facilitate the generation of pathogenic antibodies. Complement activation, a central feature of cold agglutinin-mediated hemolysis, may also be amplified in malignancy-related inflammatory states. Additionally, evolving marrow dysfunction and dysplasia preceding overt leukemia may contribute to immune dysregulation. Given the rarity of this association, these mechanisms remain speculative.

The report by Sadeghi et al. remains the clearest prior full case report describing CAS as the initial manifestation of AML [3]. Broader AML-associated immune hemolysis has also been described, although most reported cases involved warm or unspecified phenotypes [4-8]. Notably, the 2024 report by Musleh and Alhusein described concurrent AML and autoimmune hemolytic anemia with an IgG-positive direct antiglobulin test, without clear documentation of C3d positivity or cold agglutinin testing, making confirmed cold agglutinin-mediated hemolysis unlikely [8]. To our knowledge, peer-reviewed reports of AML with clearly documented cold agglutinin-mediated hemolysis are extremely limited; we identified one prior full case report meeting these criteria. Table 2 summarizes the clearly documented published cases of AML associated with cold agglutinin-mediated hemolysis.

**Table 2:** Published reports of AML-associated autoimmune hemolytic anemia and classification of cold agglutinin-mediated hemolysis Confirmed cold agglutinin-mediated hemolysis was defined as explicit documentation of CAS or cold hemolysis with supportive serologic findings; reports describing AML-associated autoimmune hemolytic anemia without adequate cold-specific characterization were classified as broader AML-associated AIHA rather than confirmed CAS. AML: acute myeloid leukemia; AIHA: autoimmune hemolytic anemia; CAS: cold agglutinin syndrome; DAT: direct antiglobulin test; IgG: immunoglobulin G; C3d: complement component 3d

Report	AML context	Hemolysis phenotype	Supporting serologic/diagnostic features	Temporal relationship to AML	Classification in this review	Key relevance
Sadeghi et al. (2025) [3]	Monocytic AML	Cold agglutinin syndrome	Reported as CAS with cold-specific hemolytic features	Initial manifestation of AML	Confirmed cold agglutinin-mediated hemolysis	Prior published case documenting CAS at AML presentation
Mangal and Buskard (1984) [4]	Acute myeloblastic leukemia	Autoimmune hemolytic anemia	AIHA reported; cold-specific serologic characterization not clearly documented	Associated with AML	Broader AML-associated AIHA; not confirmed CAS	Supports that immune hemolysis may occur in AML
Tamura et al. (1996) [5]	De novo acute myelocytic leukemia	Autoimmune hemolytic anemia	AIHA reported; cold-specific serologic characterization not clearly documented	Associated with AML	Broader AML-associated AIHA; not confirmed CAS	Additional evidence that AIHA can accompany AML
Deutsch et al. (2003) [6]	Acute myelocytic leukemia	Autoimmune hemolytic anemia	AIHA reported; cold-specific serologic characterization not clearly documented	Associated with AML	Broader AML-associated AIHA; not confirmed CAS	Historical report linking AML and AIHA
Essa et al. (2016) [7]	Acute myelomonocytic leukemia	Autoimmune hemolytic anemia	AIHA reported; cold-specific serologic characterization not clearly documented	Associated with AML	Broader AML-associated AIHA; not confirmed CAS	Supports the broader association between AML and immune hemolysis
Musleh and Alhusein (2024) [8]	Concurrent AML and AIHA	Likely non-cold AIHA	IgG-positive DAT reported; no clear C3d positivity or cold agglutinin testing documented	Concurrent with AML	Not classified as confirmed cold agglutinin-mediated hemolysis	Important recent comparator, but evidence favors a non-cold phenotype
Present case	AML, myelodysplasia-related	Cold agglutinin syndrome	C3-positive/IgG-negative DAT, cold agglutinin titer 1:320, biochemical hemolysis	Cold hemolysis preceded definitive AML diagnosis	Confirmed cold agglutinin-mediated hemolysis	Serologic improvement after rituximab did not predict hematologic recovery; AML became apparent on marrow evaluation

The prior case reported by Sadeghi et al. and the present case share a similar diagnostic challenge: both patients initially appeared to have cold agglutinin-mediated hemolysis, but subsequent bone marrow evaluation revealed AML [3]. In our case, the cold agglutinin titer declined after rituximab therapy, yet the anemia worsened and peripheral blasts emerged, underscoring that serologic improvement should not be interpreted in isolation. Earlier reports of AML-associated autoimmune hemolytic anemia help establish that immune hemolysis can occur in AML; however, most cases involved warm, mixed, or incompletely characterized phenotypes that would not meet modern criteria for CAS [4-8].

The present case highlights two important clinical messages. First, cold agglutinin-mediated hemolysis may serve as the presenting manifestation of an otherwise occult myeloid neoplasm. Second, improvement in cold agglutinin titers or other serologic markers should not be interpreted in isolation. When hematologic recovery does not occur as expected, clinicians should reassess the differential diagnosis rather than anchoring on the initial diagnosis of immune hemolysis.

More broadly, the coexistence of immune hemolysis and marrow pathology presents a diagnostic challenge, particularly in older adults with new-onset macrocytic anemia. Worsening cytopenias, persistent macrocytosis, new leukocytosis or left shift, circulating blasts, or an unexpectedly poor response to standard hemolysis-directed therapy should prompt peripheral smear review, flow cytometry when appropriate, and timely bone marrow evaluation.

## Conclusions

Cold agglutinin-mediated hemolysis may be the presenting manifestation of an occult myeloid neoplasm. In this case, an apparent serologic response to rituximab did not predict hematologic recovery, and the discordant clinical course ultimately unmasked AML. Clearly documented published AML cases with cold agglutinin-mediated hemolytic anemia remain exceedingly rare. Clinicians should pursue marrow evaluation when presumed immune hemolysis is accompanied by worsening cytopenias, persistent macrocytosis, or evolving peripheral blasts.
